# Recent Researches on Kala Azar

**Published:** 1907-08-24

**Authors:** 


### RECENT RESEARCHES ON KALA AZAR.

Captain W. S. Patton, M.B., I.M.S., has recently considerably advanced our knowledge of the extra-corporeal phases of the Leishman-Donovan body, the parasite of Kala Azar. Following up a suggestion of Rogers that the bug might act as an intermediate host for this organism, he has found all the intermediate stages of development and numerous fully developed flagellates, similar to those seen in cultures of splenic blood, in the stomachs of bugs. This interesting observation tends very strongly to incriminate the bug as the carrier or spreader of that terrible malady. It still remains to be proved how the parasites escape from the bug back to man, but the observations of Rogers as to the peculiar house spread of the disease in Assam brings further proof to support the view that some domestic insect has to do with the spread of the disease, and none is more likely in this connection than the bug.

